# Tumor Molecular Profiling in Hispanics: Moving Towards Precision Oncology and Health Equity

**DOI:** 10.1007/s40615-022-01328-0

**Published:** 2022-06-01

**Authors:** Sariemma Mendez Rodríguez, Aida M. Rodríguez-Hernández, Gabriela Torres-Torres, Hilmaris Centeno-Girona, Marcia Cruz-Correa

**Affiliations:** 1 University of Puerto Rico Comprehensive Cancer Center, San Juan, PR USA; 2 VA Caribbean Healthcare System, San Juan, PR USA; 3 PanOncology Trials, San Juan, PR USA; 4 Departments of Medicine and Biochemistry, University of Puerto Rico Medical Sciences Campus, San Juan, PR USA

**Keywords:** Precision oncology, Cancer, Somatic tumor profile, Carcinogenic pathways

## Abstract

**Background:**

Tumor molecular profiling techniques, such as next-generation sequencing (NGS) to identify somatic genetic alterations, allow physicians to have a better understanding of the affected carcinogenic pathways and guide targeted therapy. The objective of our study was to characterize common somatic alterations and carcinogenic pathways among Puerto Rican Hispanics with solid tumors.

**Methods:**

We conducted a single-institution, retrospective study to characterize molecular tumor profiles using a 592-gene NGS platform. Actionable mutations with current or developing therapies targeting affected genes/pathways were highlighted.

**Results:**

Tumors from 50 Hispanic patients were evaluated using CARIS Life Science© NGS testing. The median age of our study population was 55 (range 21–84); 54% (*n* = 27) were males. The primary tumor sites were colorectal (*n* = 24), gastric (*n* = 5), breast (*n* = 4), and lung (*n* = 3). The most common genetic mutations identified were in *TP53* (44%), *APC* (38%), and *KRAS* (32%); followed by alterations in *EGFR* (4%), *HER2* (6%), and homologous recombinant deficiency genes (*BRCA2*, 6%). Genetic alterations were found in multiple signaling pathways particularly in the cell cycle control pathway, *MAPK* and *Wnt/β-Catenin* signaling pathways. Targetable biomarkers were identified in 27/50 (54.0%) of tumors.

**Discussion:**

Molecular profiling techniques, such as next-generation sequencing, have substantially expanded access to alterations in the cancer genome. Our findings demonstrated important actionable mutations in most of the tumors evaluated and support the integration of somatic mutation profiling in the evaluation of Hispanic cancer patients with advanced cancer to help guide therapeutic options.

## Introduction

In 2021, more than 1.9 million new cancer cases are expected to be diagnosed in the USA, whereas 1660 cancer deaths per day are estimated [1]. Improvements in early detection, prevention, and treatment have reduced cancer-related incidence and mortality [2]. Tumor molecular profiling techniques, such as next-generation sequencing (NGS) to identify somatic mutations, have been instrumental to better understand tumor biology. However, the genetic signatures of tumors from non-European populations remain limited. Several studies have shown significant biological differences among different cancers across races, which play an important role in response to therapies and survival [3–6]. Studies are needed to define the genetic signatures of tumors from diverse and admixed populations such as Hispanics, to move towards guiding targeted therapy based on specific tumor profiles. Furthermore, it has been reported a lack of participation of minorities in the USA, including Hispanics, in precision oncology clinical studies [7]. This could suggest that these minority populations are not equally benefitted for targeted treatments as their Non-Hispanic Whites counterparts that comprise the majority of participants in precision oncology studies [7].

For Puerto Ricans, a Hispanic subpopulation, cancer is the leading cause of death [8]. Puerto Ricans represent a growing Hispanic population with higher incidence in mortality rates in certain cancers compared to US-Hispanic subgroups [9]. Puerto Ricans are an admixed Hispanic population with noted cancer health disparities, which underscores the importance of having a better understanding of tumor molecular profiles in order to guide precision oncology therapies to reduce cancer mortality in this Hispanic subpopulation. According to the Puerto Rico Central Cancer Registry, the five most frequently diagnosed cancers are prostate, breast, colorectal, lung and bronchus, and thyroid [10]. Prostate and breast cancer account for approximately 37.3% and 28.9% of all cancer cases among males and females, respectively, and represent the leading causes of cancer-related death. Colorectal tumors represent close to 12% of all cancers in both sexes. Lung cancer and thyroid cancer are the 3^rd^ leading cancer sites in men and women, respectively (5.6% and 11.0% of all cancers) [8, 10].

Next-generation sequencing (NGS) has become the gold standard to detect DNA mutations, copy number of variations, and gene fusions across the genome on an individual level [11]. Many of these alterations can be mapped on the known signaling pathways that control cell growth, division, cell death, and motility [12]. Despite the racial/ethnic differences in survival observed in certain cancers, there is limited data about variations in somatic mutations/alterations among diverse populations that may contribute to the observed differences [13]. There are few studies that bring attention to the fact that driver mutations in certain cancers in Hispanics differ in frequency compared to non-Hispanic Whites (NHW), which comprise the majority of the data available in the current databases and most of the individuals that have participated in clinical trials that test new therapies [14]. The underrepresentation of Hispanics in international databases can affect the interpretation of the association between genes and diseases [3, 15]. Understanding the molecular profile of Hispanics can guide optimum decision-making for adequate therapies or the development of directed treatments for cancers affecting the Puerto Rican Hispanics. Therefore, the objective of the current study was to describe the mutational profile of key genetic alterations and carcinogenic pathways on Puerto Rican Hispanic patients with solid tumors.

## Methods

### Data Sources and Study Population

A cross-sectional design was used to analyze the tumor profile from 50 Hispanics living in Puerto Rico who received care at a community oncology hospital (*Dr. Isaac Gonzalez Martinez Oncology Hospital*) in Puerto Rico from November of 2019 through July 2020. Our primary objective was to describe the predominant targetable somatic genetic alterations for malignant tumors using a 592-gene NGS panel performed by CARIS Life Sciences©. Only tumor samples were evaluated using this panel. Demographic data, clinical history, and NGS panel summary reports were obtained from the Caris Molecular Intelligence (MI) Portal. This portal is a web-based tool available for Precision Oncology Alliance (POA) providers that facilitate patient data management. Hispanics living in PR with a positive result in the NGS panel were included in the study.

### Pathway Analyses and Clinical Impact of Mutated Genes

We examined common carcinogenic pathways seen across all tumor types to identify those observed in our population using the *Kyoto Encyclopedia of Genes and Genomes (KEGG) Pathway Database* (https://www.genome.jp/kegg/)*.* This database contains a collection of pathway maps to achieve a better understanding of the biological system [16]. Additionally, we assessed the precision oncology clinical impact of the observed genetic variants using the *OncoKB* database (https://www.oncokb.org), specifically the therapeutic level 1. This level contains FDA-recognized biomarkers with a predictive response to an FDA-approved drug [17].

### Statistical Analyses

Descriptive statistics were used to characterize the dataset, using frequencies, percentages, means, and standard deviation with the statistical software STATA 15.0 (Texas).

## Results

Tumors from 50 Puerto Rican Hispanic (PRH) patients were evaluated using a NGS 592-gene panel. The median age of our study population was 55 (range 21–84); 54% were males (Table 1). The tumor sites evaluated included colorectal (*n* = 24), gastric (*n* = 5), breast (*n* = 4), lung (*n* = 3), unknown (*n* = 3), bladder (*n* = 1), bile duct (*n* = 1), pancreas (*n* = 1), endometrial (*n* = 1), ovarian (*n* = 1), hypopharynx (*n* = 1), kidney (*n* = 1), ovarian (*n* = 1), pancreas (*n* = 1), prostate (*n* = 1), spine (*n* = 1), tongue (*n* = 1), and tonsillar pillar (*n* = 1). Most patients (72%) had advanced disease (stage IV) (Table 1).

**Table 1:** Sociodemographic and clinical characteristics of study participants

Characteristic	*n*	Percentage
Sex		
Male	27	54.0%
Female	23	46.0%
Age (at sample resection)	55	
Primary tumor site		
Colorectal	24	70.6%
Gastric	5	14.7%
Breast	4	11.8%
Lung	3	8.8%
Unknown	3	8.8%
Bladder	1	2.9%
Bile duct	1	2.0%
Endometrium	1	2.0%
Hypopharynx	1	2.9%
Kidney	1	2.9%
Ovarian	1	2.9%
Pancreas	1	2.9%
Prostate	1	2.9%
Spine	1	2.9%
Tongue	1	2.9%
Tonsillar pillar	1	2.9%
Stage (at diagnosis)		
I	1	2.0%
II	2	4.0%
III	4	8.0%
IV	36	72.0%
Unknown	7	14.0%
MSI stable	50	100.0%

The most prevalent genetic mutations identified among all cancer types were in *TP53* (44%), *APC* (38%), and *KRAS* (32%). Other important genetic alterations were identified in *EGFR* (4%), *HER2* (6%), and *BRCA2* (6%). The mutations identified were found in genes that play a major role in signaling pathways, such as cell cycle control, MAPK, and *Wnt/β-Catenin* signaling, among others (Table 2). In addition, targetable mutations and/or biomarkers specific for each cancer were identified in 27/50 (54%) of all tumors (see Table 3). Only mutations for those cancers with more than 3 patients (colorectal, gastric, and breast cancers) will be discussed in detail below.

**Table 2:** Tumor mutations by signaling pathway

Signaling pathway	*n*	Percentage
Cell cycle control		
*RB1*	1	2.0%
*TP53*	22	44.0%
Chromatin remodeling/DNA methylation		
*ARID1A*	3	6.0%
*ARID2*	1	2.0%
*IDH1*	1	2.0%
*KDM6A*	1	2.0%
*KMT2C*	1	2.0%
*KMT2D*	2	4.0%
*PBRM1*	1	2.0%
*SMARCA4*	1	2.0%
DNA repair/damage		
*ATM*	3	6.0%
*BRCA2*	3	6.0%
*CCND1 amplification*	2	4.0%
*ESR1 fusion detected*	1	2.0%
*MUTYH*	1	2.0%
*POLE*	1	2.0%
*POT1*	1	2.0%
*TOP2a*	1	2.0%
*WRN*	3	6.0%
G protein signaling		
*GNAS*	1	2.0%
MAPK signaling		
*ALK fusion detected*	1	2.0%
*AR*	4	8.0%
*BRAF*	1	2.0%
*EGFR amplification*	1	2.0%
*EGFR mutation*	1	2.0%
*ER*	5	10.0%
*ERBB2 (Her2/Neu) amplification*	2	4.0%
*ERBB2 (Her2/Neu) mutation*	1	2.0%
*ERBB3*	1	2.0%
*FGF3 amplification*	1	2.0%
*FGF4 amplification*	1	2.0%
*KIT*	3	6.0%
*KRAS*	16	32.0%
*NF1*	1	2.0%
*NRAS*	1	2.0%
*PR*	2	4.0%
*ROS1 fusion detected*	1	2.0%
*SF3B1*	1	2.0%
*VHL*	1	2.0%
Micro RNA biogenesis pathway		
*DICER1*	1	2.0%
NF-kB signaling pathway		
*NFKBIA amplification*	1	2.0%
NRF pathway		
*NFE2L2*	1	2.0%
PI3K/Akt signaling pathway		
*PIK3CA*	7	14.0%
*FBXW7*	3	6.0%
*KIT*	2	4.0%
*MCL1 amplification*	1	2.0%
*PTEN*	3	6.0%
*RET*	2	4.0%
RNA splicing		
*SF3B1*	1	2.0%
TGFβ signaling		
*SMAD2*	1	2.0%
Wnt/β-catenin signaling pathway		
*APC*	19	38.0%
*CDC73*	1	2.0%
*CD274 (PD-L1) amplification*	1	2.0%
*PDL1 positive*	10	20.0%
*E-cadherin (CDH1)*	2	4.0%
*RNF43*	1	2.0%

**Table 3:** Targetable mutations and/or biomarkers by specific cancer type found in PRH tumors

Cancer type	Targetable mutations and/or biomarkers
CRC	*BRAF*, *KRAS*, *NRAS*
Lung	*EGFR*, *ROS1* fusions
Cholangiocarcinoma	*IDH1*

### Colorectal Cancer

**Fig. 1:**
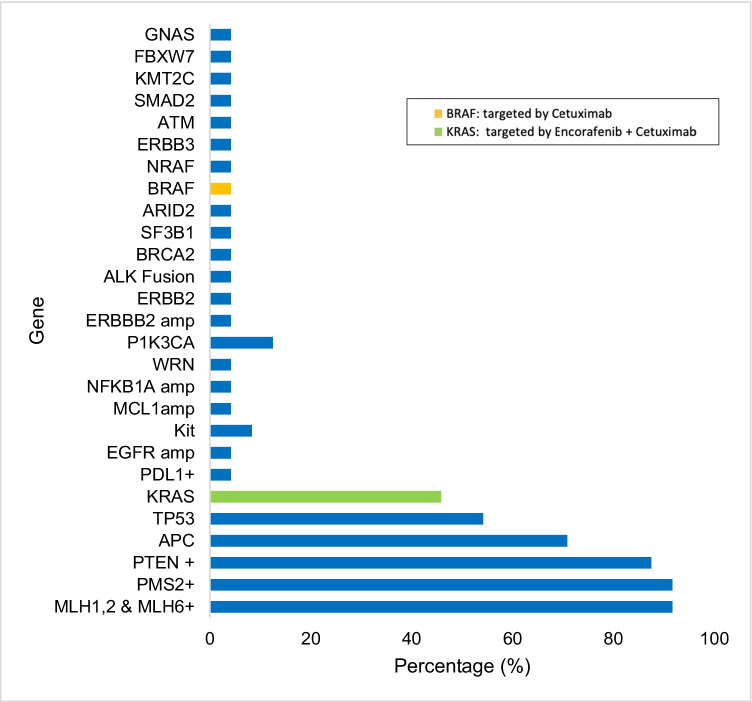
Frequencies of molecular alterations and targetable genes with available therapies among colorectal tumors (*n* = 24)

Table 4 shows the mutated genes found for CRC tumors from PRH. Most colorectal cancer genetic alterations found were mutations in tumor suppressor genes *APC* (70.8%) and *TP53* (54.2%), *KRAS* (45.8%), and *PIK3CA* (16.7%). Most of the mutations found in *APC* were truncating mutations (55.6% were nonsense and 22.2% were frameshift mutations) and 22.2% were unknown mutations. For *TP53*, 63.6% of the mutations were missense, 27.3% were frameshift, and 0.9% were nonsense. All the mutations found in *KRAS* gene were missense mutations. Lastly, 50% of the mutations for *PIK3CA* were missense and 50% were unknown. *BRCA2* (4.2%), *EGFR* (4.2%), and *ERRB2* (4.2%) were among the less common mutated genes in CRC. All of the colorectal tumors studied were microsatellite stable (Fig. 1; *n* = 24).

**Table 4:** Mutated genes found in CRC tumor samples from PRH (*n* = 24)

Gene	Mutated samples	Frequency (%)
*APC*	17	70.8%
*TP53*	13	54.2%
*KRAS*	11	45.8%
*PIK3CA*	4	16.7%
*ERBB2* amplification	2	8.3%
*KIT*	2	8.3%
*ALK* fusion detected	1	4.2%
*ARID1A*	1	4.2%
*ARID2*	1	4.2%
*ATM*	1	4.2%
*BRAF*	1	4.2%
*BRCA2*	1	4.2%
*EGFR*	1	4.2%
*ERBB2* mutation	1	4.2%
*ERBB3*	1	4.2%
*FBXW7*	1	4.2%
*GNAS*	1	4.2%
*KMT2C*	1	4.2%
*MCL1* amplification	1	4.2%
PDL1-positive	1	4.2%
*NFKBIA* amplification	1	4.2%
*NRAS*	1	4.2%
*PTEN*	1	4.2%
*SF3B1*	1	4.2%
*SMAD2*	1	4.2%
*WRN*	1	4.2%

### Gastric Cancer

**Fig. 2:**
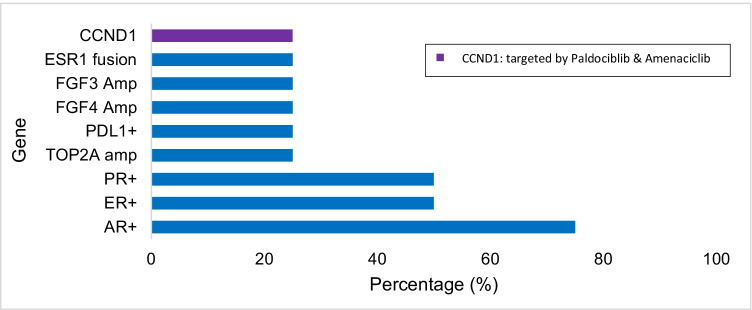
Frequencies of molecular alterations and targetable genes with available therapies among breast tumors (*n* = 4)

Table 5 shows the gene alterations found in gastric cancer samples. The most common mutations in gastric tumors (*n* = 5) were detected in *TP53* (60%), *CDH1* (40%), and *PIK3CA* (20%) (Fig. 2). For *TP53*, 33.3% of the mutations were nonsense, 33.3% were missense, and 33.3% were intronic mutations. Fifty percent of the mutations found in *CDH1* were frameshift mutations and 50% were deletions.

**Table 5:** Mutated genes found in gastric cancer tumor samples from PRH (*n* = 5)

Gene	Mutated samples	Frequency (%)
*PDL1-*positive	5	100.0%
*TP53*	3	60.0%
*CDH1*	2	40.0%
*APC*	1	20.0%
*BRCA2*	1	20.0%
*CDC73*	1	20.0%
*PIK3CA*	1	20.0%
*PTEN*	1	20.0%

### Breast Cancer

**Fig. 3:**
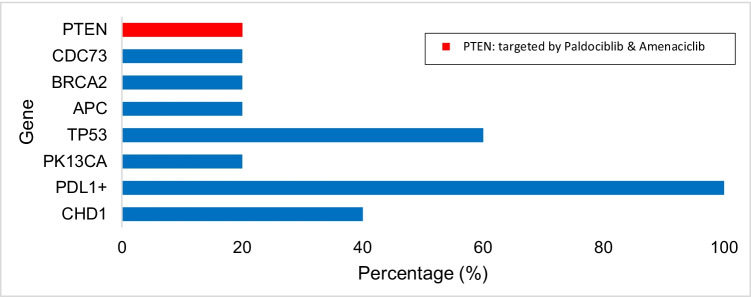
Frequencies of molecular alterations and targetable genes with available therapies among gastric tumors (*n* = 5)

Table 6 shows the gene alterations found in breast cancer samples. Among patients with breast cancer (*n* = 4), 75% had mutations in *AR*, 50% in *ER*, and 50% in *PR*. An *ESR1* fusion and *PDL1* expression were identified in 1 patient, respectively (Fig. 3). No alterations in key actionable genes including *ERBB2* (HER2), *NTRK* fusions, or *PIK3CA* mutations were identified in breast cancer patients.

**Table 6:** Mutated genes found in breast cancer tumor samples from PRH (*n* = 24)

Gene	Mutated samples	Frequency (%)
AR positive	3	75.0%
PR positive	2	50.0%
ER positive	2	50.0%
CCND1 amplified	1	25.0%
ESR1 fusion detected	1	25.0%
FGF3 amplification	1	25.0%
FGF4 amplification	1	25.0%
PDL1 positive	1	25.0%
Top2A amplification	1	25.0%

## Discussion

Cancer is the leading cause of death among US-Hispanics and the 2^nd^ most common cause of death in Puerto Rico [1]. Unequal access to healthcare and a high prevalence of major cancer risk factors, such as obesity and diabetes, are among the many reasons why cancer continues to be a major public health concern among Hispanics [18]. To move towards achieving cancer health equity, the tumor genetic landscape needs to be characterized in diverse minority populations, such as Hispanics, to inform precision oncology strategies and reduce cancer burden [19]. In this study, we report mutation profiles of solid tumors from PRH, a Hispanic subpopulation, and detected genetic alterations involved in multiple pathways, including those with currently available and potential therapeutic targets.

### Colorectal Cancer (CRC)

Most CRC are due to chromosomal instability, which results in the activation of oncogenes (e.g., *KRAS* from the *PI3K-AKT* signaling pathway) and inactivation of tumor suppressor genes (e.g., *p53* and *APC*) [20, 21]. As shown in the present study, most CRC alterations found were in *APC* (71%), *TP53* (54%), and oncogene *KRAS* (45%). In addition, *TP53*, *APC*, and *KRAS* mutations are also seen in breast, gastric, and lung cancers, which may explain in part the high prevalence of genetic alterations in these three genes [22].

Previous studies have highlighted differences in incidence of cancer driver mutations according to race/ethnicity [23]. Recent data from our group evaluating 24 patients with CRC shown that most colorectal tumors were microsatellite stable (98%), CIMP-low (92%), and had wild-type *KRAS* (69%) and *BRAF* (91%) [24]. Another study examining mutational profiles among PRH with CRC reported similar mutation rates for *KRAS* (39%), with the highest frequency of mutations in codon 12 (12 Asp (39.5%) and 12 Val (25%)) [25]. In the current study, among the 16 patients with *KRAS* mutations (combined tumors), the two most frequent mutations were in exon 12 (43%) and 13 (25%) with G12D and G13D, respectively. *KRAS* mutations located in codon 12 (G12C) were found in 6% of tumors profiled, which is significant as we now have targeted therapy for patients whose tumors have *KRAS* G12C mutations [26].

Another important biomarker secondary to defects in the mismatch repair pathway is microsatellite instability (MSI) and guides treatment options for CRC patients [27]. MSI predicts response to checkpoint inhibitors such as anti-PDL1 and anti-CTL4 therapies [28]. In our study population, all CRC tumors were microsatellite stable, which is in contrast with higher prevalence of MSI-tumors reported in other racial/ethnic groups. MSI rates among non-Hispanics Blacks, US Hispanics, and non-Hispanic Whites have been reported to be 12%, 12%, and 14%, respectively [29]. Interestingly, an *ALK*-fusion gene was detected among one of the colorectal tumors in our study. This gene fusion is rare and is identified in 0.5–2.5% of patients with CRC [30].

### Gastric Cancer

There are two distinct subtypes of gastric cancer, intestinal and diffuse type [20, 31]. Pathways altered in the intestinal type gastric cancer include alterations in p53 signaling pathway, Wnt/β-Catenin signaling pathway, *PI3K/AKT* signaling pathway, and TGFβ signaling pathway [32]. Mutations in the *CDH1*, which is associated with the *MAPK* signaling pathway, are commonly detected in diffuse type gastric cancer [33]. Among the gastric tumors of our Hispanic cohort, the most common mutations identified were on *TP53* (60%), *CDH1* (40%), and *PIK3CA* (20%) (Fig. 3). Our findings are similar to previously reported mutations in gastric cancer among non-Hispanic patients, with the least common mutation being found at *PIK3CA*. This was the third most common gene mutated among the PRH gastric tumors examined, which is associated with tumor aggressiveness [32, 34]. Of note, all of the gastric tumors studied had PD-L1 over-expression. PD-L1 tumor expression has been reported to be an important prognostic predictor for positive response to immunotherapy [35].

### Breast Cancer

Around 70% of breast cancers express estrogen receptors (ER +) [36]. Mutations in *HER2* and *ER* are common in non-Hispanic breast tumors (28%) [26]. Additional key pathways such as the Notch signaling pathway and the *Wnt* signaling pathways play an important role in breast carcinogenesis [26]. Among the PRH patients with breast cancer evaluated in this study, 75% were found to have genetic alterations in *AR*: 50% in *ER* and 50% in *PR*; additional pathways identified included *PI3K-AKT*, *MAPK*, and *p53* signaling pathways. Interestingly, among the tumors evaluated, we detected *PDL-1* expression in one patient and an *ESR1* fusion in another. Clinical studies have demonstrated how mutations in *ESR1* are frequently associated with poor prognosis and metastasis, related to hormone-resistant breast cancer, and is targeted by several therapeutic agents [36]. Frequently, fusions or missense mutations of the ESR1 gene have been found to be involved in metastatic progression [36]. A larger number of breast tumors from PRH women are needed to accurately describe the somatic mutational profile among this Hispanic subgroup.

The development of novel therapies targeting specific pathways requires a comprehensive understanding of somatic mutational profiles to inform precision medicine and improve therapeutic responses. In our sample population, 86% of all tumors harbored a targetable mutation and/or biomarker. Currently, there are multiple targeted therapies, such as *MEK* and *ERK* inhibitors for tumors with *KRAS* mutations [37]. Moreover, androgen receptor inhibitors and *HER2* inhibitors may be used against different tumor types, including breast, colon, and gastric cancer [38]. As immunotherapy with check point inhibitors continues to evolve, patients with *PDL-1* overexpression may benefit from this treatment strategy [28].

Limitations that may affect our study include the fact that it is a single-institution study from a community-based oncologic hospital and may not be representative of the PRH population living across the island. For this reason, comparisons with other Hispanic populations, such as the subset of Hispanics of the AACR GENIE (Genomics Evidence Neoplasia Information Exchange) project, were not performed. Nonetheless, this study serves as a baseline for future studies including larger sample sizes for each type of cancer. In addition, details about previous chemotherapy and/or treatment given prior or after tumor analysis, and information on social determinants of health (e.g., education, economic status, and environmental exposures) and past medical history were not available. Other studies have reported that tumor expression can be affected by certain therapies. For example, after a patient receives a thiopurine drug therapy, the tumor can express other mutations that create resistance to therapy. This highlights the importance of documenting the time point of the NGS testing. In future studies, the inclusion of patient clinicopathological characteristics will allow adjusting the analysis for potential confounders that may affect our population’s mutational status [3, 39].

When analyzing tumor-only specimens, there is a chance of introducing variability to the results due to therapy-related changes, the purity of the tumor, and sample collection methods. Additionally, there must be a careful selection when deciding how to discriminate somatic from germline variants. However, tumor-only sequencing has the advantage of being cost-effective, helping inform a diagnostic, predict prognosis for certain tumors, and providing tumor profiling for mutational burden [40, 41].

The majority of our sample biopsies were taken from primary tumor site locations; however, in 8.8% of the samples, the tumor site location was unknown. Nevertheless, this study presents robust, clinical data on somatic genetic alterations for an underserved, Hispanic subpopulation and demonstrates a high prevalence of targetable molecular tumor biomarkers. Thus, efforts are needed to educate the medical and surgical community to incorporate NGS testing for management of advanced cancer among Hispanics. Incorporating precision oncology will require education across all levels of the medical and general community, access to clinical molecular tumor profiling, and health policy efforts. Inclusion of diverse populations in biomarker-specific oncology clinical trials will further promote precision oncology and increase health equity among diverse populations.
